# Ultraslow mid-ocean ridges: ultra focused magma supply

**DOI:** 10.1093/nsr/nwaf101

**Published:** 2025-03-19

**Authors:** Jian Lin, Fan Zhang, Zhiyuan Zhou

**Affiliations:** Advanced Institute for Ocean Research and Department of Ocean Science and Engineering, Southern University of Science and Technology, China; State Key Laboratory of Tropical Oceanography, South China Sea Institute of Oceanology, Chinese Academy of Sciences, China; State Key Laboratory of Tropical Oceanography, South China Sea Institute of Oceanology, Chinese Academy of Sciences, China; Advanced Institute for Ocean Research and Department of Ocean Science and Engineering, Southern University of Science and Technology, China

The mid-ocean ridge (MOR) is formed by the divergence of tectonic plates, the process of which shapes Earth's surface. Mantle melting beneath global MORs is a fundamental process that drives mantle flow, controls oceanic crustal accretion, and influences global geochemical cycles. Melts migrate from the mantle to the surface of Earth, yet their transport and distribution along MORs, as well as their impact on crustal construction, remain subjects of debate.

Initially, scientists focused primarily on variations in plate spreading across the ridge axis, assuming that they were 2D structures. However, they later recognized that along-axis variations are also crucial [1]. Ultraslow-spreading centers are geologically unique environments in which new oceanic crust is formed at extremely slow rates, exhibiting significant along-axis variability in volcanic and tectonic activities [2]. The Gakkel Ridge in the Arctic Ocean, with a full spreading rate of 7–14 mm/yr, presents an unparalleled natural laboratory for investigating the melt-migration process under ultraslow-spreading conditions.

During the groundbreaking Joint Arctic Scientific Mid-Ocean Ridge Insight Expedition (JASMInE) in 2021, a Chinese scientific team conducted a comprehensive geophysical survey. Using an ocean-bottom seismometer (OBS) array at the eastern segment of the Gakkel Ridge, they uncovered an extremely abundant and highly variable magma supply and demonstrated a new mechanism of active mantle upwelling [3]. This extraordinary success has revolutionized our understanding of mantle dynamics at spreading centers. Yet, several critical questions remain enigmatic, such as the segment-scale variations in magma supply.

**Figure 1.**
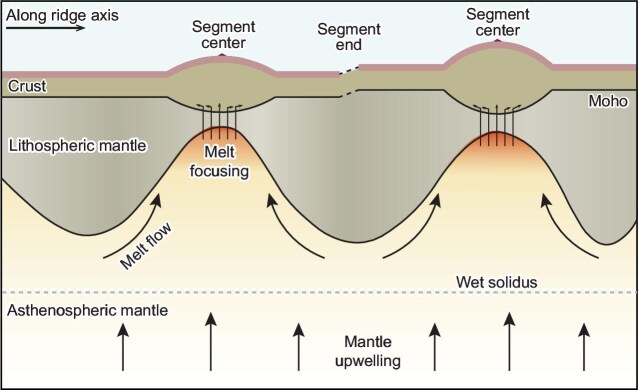
Schematic model illustrating the along-ridge melt focusing and generation. Due to more robust magma supply, the segment centers are associated with thinner lithosphere and thicker crust. Meanwhile, the segment distal ends exhibit thicker lithosphere and thinner crust.

The JASMInE project utilized magnetotelluric (MT) methods, which are primarily sensitive to the presence of melt. By linking mantle melting with variations in electrical resistivity, Zhang *et al.* [4] provide direct geophysical evidence for a model that shows how melt is distributed beneath ultraslow-spreading ridges. The findings indicate that segment centers have thinner lithosphere due to repeated magma intrusions. Melt accumulates in relatively shallow zones (20–45 km depth), sustaining localized magmatic activity. In contrast, thicker lithosphere is observed at segment distal ends, where deep valleys form and the underlying melting zone is missing. They further proposed that, in ultraslow-spreading systems, melts tend to migrate toward segment centers and create localized zones of crustal accretion (Fig. 1).

The observed highly variable lithospheric thickness along the Gakkel Ridge contrasts with a microseismicity study in the ultraslow-spreading Southwest Indian Ridge, which shows an approximately constant lithospheric thickness under both the magmatic and nearly-amagmatic ridge segments [5]. This discrepancy indicates complicated thermal structure evolution and melting migration processes of ultraslow-spreading ridge systems.

Nevertheless, these new findings are consistent with the observed highly variable crustal thickness along the Gakkel Ridge. The most likely scenario is that, in regions where the lithosphere is thin, the mantle can ascend adiabatically to shallower depths, resulting in a higher degree of melting. Meanwhile, the limited melt that is generated at regions of thicker lithosphere migrates upward along the permeability barrier at the base of the lithosphere.

Furthermore, the mantle beneath the segment center experiences a higher degree of partial melting and leaves a more depleted residual mantle, which is poorer in incompatible elements and volatiles. In contrast, the mantle beneath the segment ends remains more fertile and may retain a higher potential for future melting. Overall, uneven melt distribution at MORs leads to long-term geochemical and structural variations in the mantle, influencing both past and future mantle dynamics.

Despite advances in imaging of the along-ridge electrical structure, integrated approaches, including high-resolution geophysical observations, improved mantle flow models and new geochemical constraints from oceanic crust and abyssal peridotites, are still needed to unravel the complexities of MOR lithosphere and melting processes.
